# Respiration and the watershed of spinal CSF flow in humans

**DOI:** 10.1038/s41598-018-23908-z

**Published:** 2018-04-04

**Authors:** Steffi Dreha-Kulaczewski, Mareen Konopka, Arun A Joseph, Jost Kollmeier, Klaus-Dietmar Merboldt, Hans-Christoph Ludwig, Jutta Gärtner, Jens Frahm

**Affiliations:** 1 Department of Pediatrics and Adolescent Medicine, Division of Pediatric Neurology, University Medical Center Göttingen, 37075 Göttingen, Germany; 2 School of Medicine, University Medical Center Göttingen, 37075 Göttingen, Germany; 3 Biomedizinische NMR Forschungs GmbH am Max-Planck-Institut für biophysikalische Chemie, 37077 Göttingen, Germany; 4 DZHK (German Center for Cardiovascular Research), partner site Göttingen, Göttingen, Germany; 5 Department of Neurosurgery, Division of Pediatric Neurosurgery, University Medical Center Göttingen, 37075 Göttingen, Germany

## Abstract

The dynamics of human CSF in brain and upper spinal canal are regulated by inspiration and connected to the venous system through associated pressure changes. Upward CSF flow into the head during inspiration counterbalances venous flow out of the brain. Here, we investigated CSF motion along the spinal canal by real-time phase-contrast flow MRI at high spatial and temporal resolution. Results reveal a watershed of spinal CSF dynamics which divides flow behavior at about the level of the heart. While forced inspiration prompts upward surge of CSF flow volumes in the entire spinal canal, ensuing expiration leads to pronounced downward CSF flow, but only in the lower canal. The resulting pattern of net flow volumes during forced respiration yields upward CSF motion in the upper and downward flow in the lower spinal canal. These observations most likely reflect closely coupled CSF and venous systems as both large caval veins and their anastomosing vertebral plexus react to respiration-induced pressure changes.

## Introduction

The compartments of the human CSF system are closely interconnected comprising the internal ventricular system, the subarachnoid spaces embedding the brain and the spinal cord. In particular, the subarachnoid spaces of brain and spine communicate freely via the foramen magnum at the cranio-cervical junction. In contrast to intracranial and upper cervical spinal CSF flow, few studies have addressed CSF dynamics along the entire spine. Moreover, diverse results have raised controversy and clinically relevant mechanisms of CSF flow in the spinal canal are still poorly understood. Thus, the pathogenesis of spinal cord developmental disorders like syringomyelia is vastly unknown.

Using radionuclide scintiphotographic techniques^1–3^ Di Chiro *et al*. described both a spinal ascent and in a later study a descent of CSF, which led to the postulation of a two-directional spinal CSF flow^3^. These results could not always be reproduced and other authors explained their pattern of tracer accumulation and uptake by pulsatile flow and mixing of CSF^4^. Early ECG-synchronized cine flow magnetic resonance imaging (MRI) studies also suggested a pulsatile nature of spinal CSF flow without net movement^5^ or reported systolic-diastolic flow until the thoracolumbar junction but none below in lumbar regions^6^. Cardiac-related CSF oscillations were also measured in cervical segments of the spine with respiration-induced bulk flow superimposed and moving in separate channels, i.e. downward during inspiration and upward during expiration^7^. Henry-Feugeas *et al*.^8^ also referred to separate dynamic channels in the spinal CSF space with different flow directions.

Spinal and intracranial vascular pulsations were considered as the main factors initiating motion with cardiac pulsation most prevalent at cervical levels and increasing influence of respiration in thoracic and lumbar regions^9^. Homogeneous cardiac-driven CSF flow in the cervical spine was noticed by 4D cardiac-gated phase-contrast flow MRI^10^.

In recent real-time MRI studies we identified forced inspiration as the main driving force of CSF flow in humans^11,12^. A consistent upward surge of CSF towards the brain was observed in response to deep inspiration in the aqueduct and at cervical level C3 and for the majority of measurements at upper and middle thoracic levels. Concomitant analyses of cerebral venous blood flow in epidural veins demonstrated enhanced downward flow of venous blood out of the head during deep inspiration which was counterbalanced by the simultaneous upward movement of CSF^12^. Here, we address for the first time CSF dynamics along the entire spinal canal using real-time phase-contrast flow MRI at high spatial and temporal resolution.

## Results

### Real-time flow MRI

**Figure 1:**
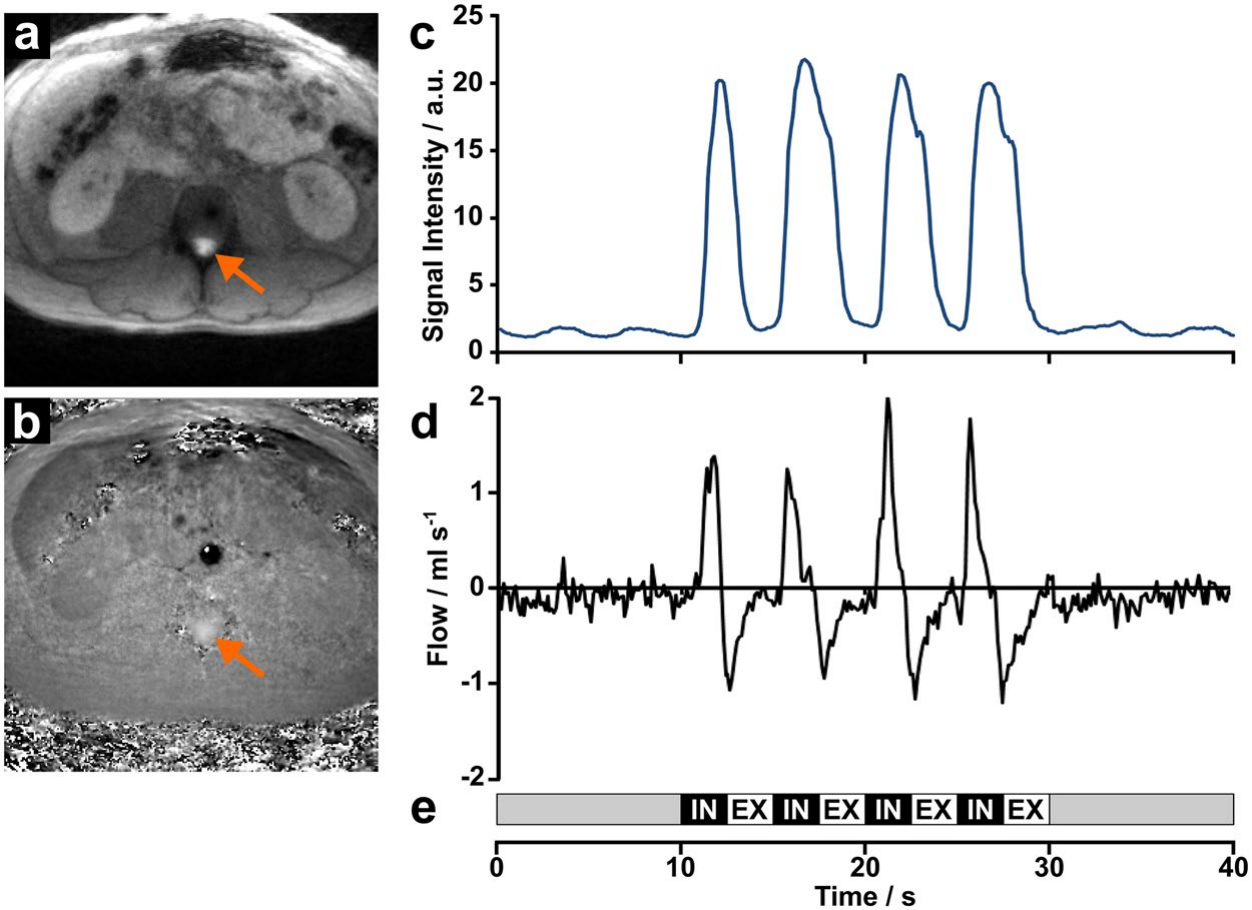
Real-time phase-contrast flow MRI of the spinal canal. (**a**) Selected anatomic image at lumbar level L4 depicting CSF through-plane flow (arrow) and (**b**) corresponding velocity map with bright signals (arrow) indicating CSF flow in upward direction. (**c**) Signal intensity in the abdominal wall from anatomic images indicating the subjects breathing performance and (**d**) corresponding CSF flow (ml s^−1^) in response to (**e**) a visually presented breathing protocol comprising 4 cycles of 2.5 s forced inspiration (IN) and 2.5 s expiration (EX).

Figure 1 shows a typical result of a real-time phase-contrast flow MRI study of the spinal cord at lumbar level L4 in supine position. The serial MRI data consist of simultaneous pairs of an anatomic image (Fig. 1a) and a corresponding velocity map (Fig. 1b). Here, the selected example refers to inspiration. A region-of-interest (ROI) for analysis was defined in anatomic images which exhibit a high sensitivity to through-plane flow (bright signal in Fig. 1a, arrow). The quantitative analysis of directional flow velocities (bright upward signal in Fig. 1b, arrow) yields time courses of flow velocities (not shown) and flow rates (Fig. 1d) in response to the 40 s breathing protocol (Fig. 1e). The actual performance of the subject is visualized by MRI signal intensity variations of the abdominal wall, which are synchronous with respiration (Fig. 1c) and taken from the same real-time image series. The results reveal a distinct positive CSF flow, which accompanies every onset of forced inspiration and refers to an upward surge of the fluid. This finding is in concordance with our previous results at the aqueduct and upper spinal canal^12^. Forced expiration, on the other hand, results in a reversal of flow direction and hence in a downward movement. The observation of a superimposed low-amplitude component must be ascribed to cardiac-related pulsations.

### CSF flow along the spinal canal

**Figure 2:**
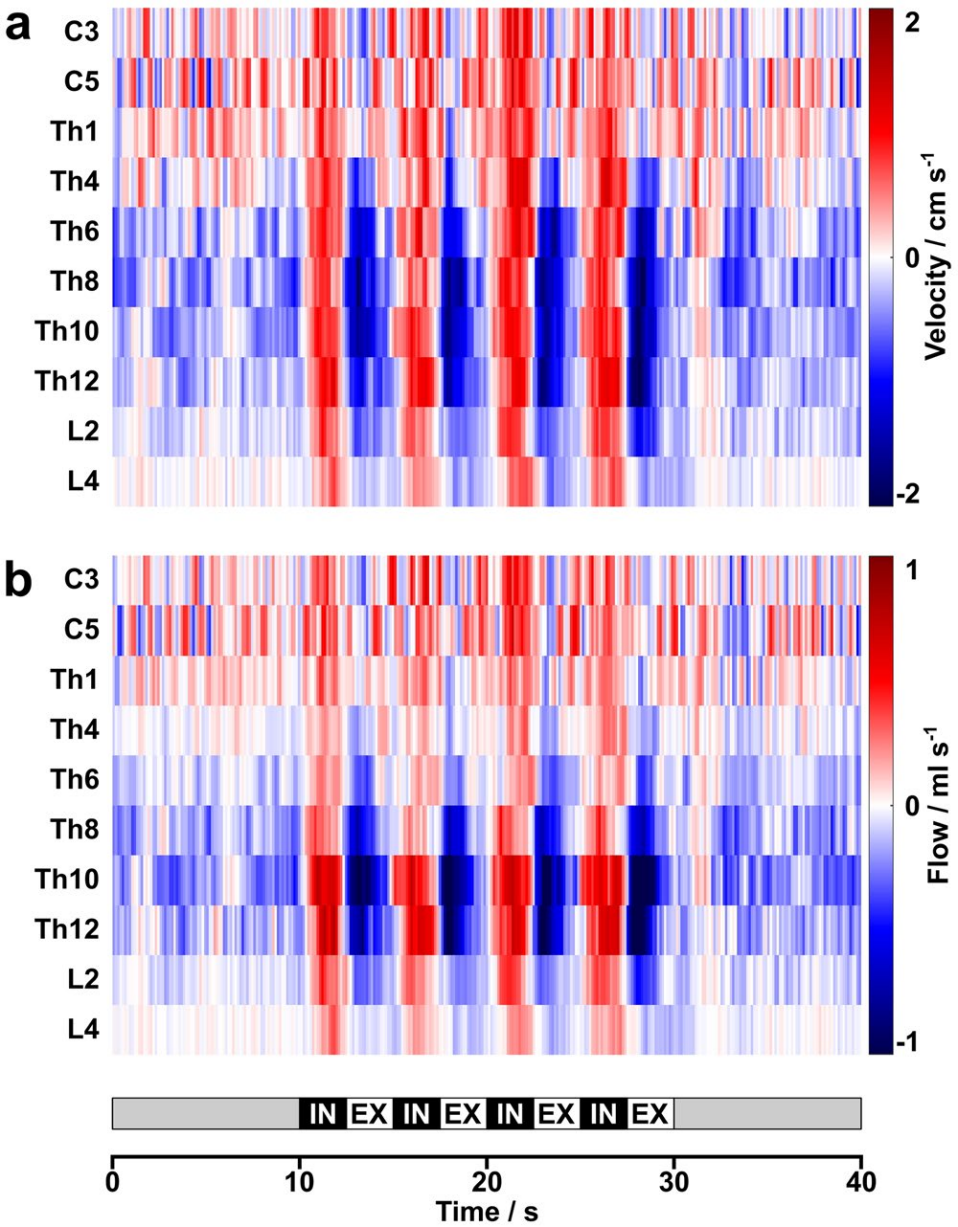
CSF dynamics along the spinal canal. (**a**) Mean color-coded flow velocities and (**b**) flow rates averaged across subjects as a function of spinal level. CSF flow increases in upward direction (red) with every forced inspiration at all spinal levels. During subsequent expiration CSF dynamics follow a bidirectional pattern with very low flow in the upper spinal canal and a pronounced downward movement (blue) in the lower spinal canal. White color represents zero flow.

**Figure 3:**
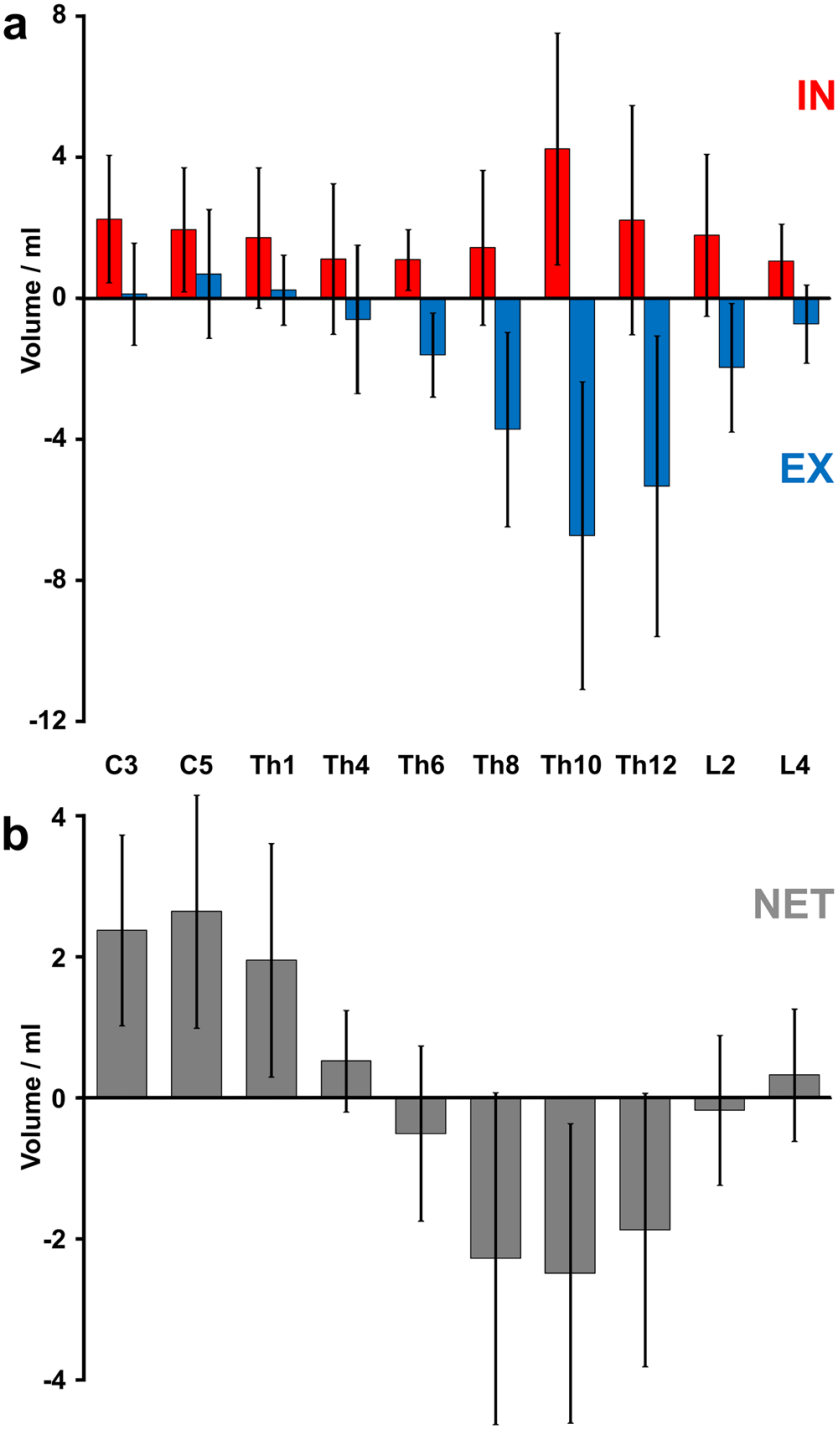
Watershed of spinal CSF flow during forced breathing. (**a**) Mean CSF volumes averaged across subjects from all 4 cycles of forced inspiration (IN = red) and expiration (EX = blue) as a function of spinal level. While inspiratory CSF volumes represent upward flow in the entire spinal canal, expiration leads to a downward directionality in the lower spinal canal. (**b**) Corresponding net CSF flow volumes point upwards at cervical and high thoracic levels and downwards between mid and lower thoracic regions. Error bars represent standard deviations.

The central findings for CSF dynamics along the entire spinal canal are summarized in Figs. 2 and 3 for 19/20 subjects. One subject failed to comply with the breathing protocol and in 10/19 subjects a total of 18/190 measurements could not be analyzed due to technical reasons leading to impaired image quality.

Figure 2 shows color-coded results for the mean flow velocity (Fig. 2a) and flow rate (Fig. 2b) averaged across subjects. Both datasets indicate cranially directed (red) CSF movements with every forced inspiration at all spinal levels. During exhalation CSF follows a caudal movement (blue), which is most prominent in lower thoracic regions, while upper thoracic and cervical levels do not exhibit a clear trend. The 10 s periods of normal breathing before and after the 4 cycles of forced breathing reveal similar patterns though at reduced strength.

Figure 3 depicts the CSF flow volumes (Fig. 3a) for 4 cycles (20 s) of 2.5 s forced inspiration and 2.5 s expiration. Inspiration caused consistent positive values (upward flow) along the entire spinal canal with the highest flow volumes at Th10. In contrast, expiratory CSF flow volumes were rather small at cervical and upper thoracic levels, but revealed a distinct downward flow in the lower spinal canal, in particular at levels Th6 to L2. Consequently, the resulting net CSF flow volumes for 20 s of forced respiration shift directionality over the length of the spinal canal (Fig. 3b), yielding a watershed at about the level of the heart. In other words, the net CSF flow volumes were positive from C3 to Th1, implying an upward movement towards the skull, whereas net flow volumes at levels Th8 to Th12 moved downward towards the thecal sac below the conus of the spinal cord. For all 19 subjects the quantitative CSF flow volumes during inspiration and expiration as well as the net volumes are summarized in Table 1.

**Table 1:** Net CSF volumes (ml) at all spinal levels in response to forced breathing.

Subject		#1	#2	#3	#4	#5	#6	#7	#8	#9	#10	#11	#12	#13	#14	#15	#16	#17	#18	#19	Mean^1^ ± SD
C3	In	2.6	1.6	1.6	−1.2	4.4	0.4	4.0	1.2	0.8	2.2	0.8	1.2	2.4	n.a.	1.2	3.6	5.2	n.a.	5.6	2.4 ± 1.8
	Ex	−1.2	−0.1	0.4	3.2	−0.8	−0.1	−1.6	0.4	0.4	1.4	0.8	2.0	−1.2		0.0	1.6	−0.8		−2.4	0.1 ± 1.6
	Net	1.4	1.6	2.2	2.4	4.0	0.4	2.2	1.6	1.2	3.6	1.6	3.2	1.4		1.2	5.4	4.4		3.2	2.4 ± 1.2
C5	In	5.6	2.4	1.0	0.8	4.8	1.2	2.0	0.0	2.6	n.a.	0.4	1.2	0.8	n.a.	0.6	4.0	3.2	3.2	−0.4	2.0 ± 1.6
	Ex	−2.4	0.8	−0.1	2.4	−0.4	2.0	−0.4	0.2	1.2		0.4	1.2	−0.2		0.8	1.6	0.1	−1.2	6.0	0.8 ± 1.8
	Net	3.6	2.8	0.8	3.2	4.0	2.8	2.0	0.2	4.0		0.6	2.2	0.8		1.6	5.6	3.2	1.6	5.6	2.8 ± 1.6
Th1	In	8.6	1.5	1.2	0.2	3.4	0.4	2.0	n.a.	2.4	0.3	0.4	0.2	0.8	0.7	1.2	1.8	1.8	0.9	3.2	1.7 ± 2.0
	Ex	−2.1	−0.5	0.0	0.9	−1.0	1.1	−0.1		1.2	0.2	1.4	0.7	0.2	−0.1	1.7	0.4	−0.8	−0.4	1.9	0.2 ± 1.0
	Net	6.5	1.0	1.2	1.1	2.4	1.5	1.8		3.6	0.4	1.3	0.8	1.1	0.6	2.9	2.2	1.1	0.5	5.1	2.0 ± 1.6
Th4	In	2.5	0.3	0.2	0.2	2.3	0.4	3.9	0.2	0.5	4.9	0.6	0.1	0.9	0.8	2.7	2.6	3.2	0.0	−5.2	1.1 ± 2.1
	Ex	−1.5	−0.2	0.0	0.1	−2.0	−0.2	−4.2	0.1	−0.7	−3.4	−0.2	0.0	−0.8	−0.3	−1.1	−2.2	−0.9	−0.3	6.5	−0.6 ± 2.1
	Net	1.0	0.1	0.3	0.3	0.4	0.2	−0.3	0.4	−0.2	1.6	0.4	0.2	0.1	0.4	1.6	0.5	2.4	−0.4	1.3	0.5 ± 0.7
Th6	In	3.3	1.4	0.6	−0.4	1.9	0.5	0.8	0.6	0.8	1.6	0.9	0.4	1.4	1.0	0.9	1.5	2.6	0.1	1.2	1.1 ± 0.8
	Ex	−2.3	−0.6	−0.9	−1.0	−1.8	−1.8	−1.1	−2.2	−1.2	−4.6	−0.6	−1.0	−1.0	−2.0	−3.0	−3.5	−1.5	−0.6	0.6	−1.6 ± 1.2
	Net	1.0	0.8	−0.4	−1.4	0.0	−1.3	−0.4	−1.7	−0.4	−3.1	0.3	−0.5	0.4	−1.0	−2.1	−2.0	1.1	−0.6	1.7	−0.5 ± 1.2
Th8	In	3.3	2.5	0.5	−0.7	3.3	1.3	1.6	0.8	1.4	−1.5	0.9	0.4	1.6	0.2	0.6	1.3	9.0	−0.2	1.2	1.4 ± 2.2
	Ex	−4.0	−2.9	−1.2	−1.1	−8.3	−2.1	−8.7	−6.0	−2.7	−6.2	−1.6	−1.1	−2.0	−1.8	−2.8	−5.0	−9.2	−0.5	−3.1	−3.7 ± 2.8
	Net	−0.8	−0.4	−0.6	−1.8	−5.0	−0.8	−7.2	−5.3	−1.4	−7.7	−0.7	−0.6	−0.4	−1.6	−2.2	−3.6	−0.2	−0.7	−2.0	−2.3 ± 2.4
Th10	In	8.8	3.8	0.8	1.6	7.1	6.4	4.5	11.0	1.7	9.1	2.3	1.0	2.7	0.2	3.0	3.1	5.4	0.3	7.6	4.2 ± 3.3
	Ex	−16.7	−2.6	−3.9	−3.4	−10.6	−10.3	−5.3	−15.6	−3.4	−10.8	−3.2	−5.6	−6.0	−0.8	−3.9	−7.4	−7.2	−3.5	−7.3	−6.7 ± 4.4
	Net	−7.9	1.2	−3.0	−1.9	−3.5	−4.0	−0.8	−4.5	−1.7	−1.7	−0.9	−4.6	−3.3	−0.6	−0.9	−4.2	−1.8	−3.3	0.3	−2.5 ± 2.1
Th12	In	6.6	2.4	n.a.	0.3	n.a.	5.8	1.6	12.1	2.1	3.3		1.8	1.2	0.6	n.a.	n.a.	4.8	n.a.	2.2	2.2 ± 3.2
	Ex	−12.4	−3.6		−3.2		−5.5	−2.2	−14.7	−4.3	−8.7	n.a.	−2.1	−0.9	−1.8			−6.7		−2.8	−5.3 ± 6.4
	Net	−5.8	−1.2		−2.9		0.2	−0.6	−2.6	−2.2	−5.4		−0.4	0.3	−1.3			−1.9		−0.6	−1.9 ± 1.9
L2	In	1.7	0.9	−0.2	2.0	n.a.	5.0	1.2	7.8	1.4	3.2	0.5	−0.1	−0.2	n.a.	n.a.	n.a.	3.5	−0.7	0.8	1.8 ± 2.3
	Ex	−2.0	−1.2	−0.1	−0.4		−4.2	−2.0	−5.6	−1.7	−4.9	−1.2	0.3	−0.6				−3.8	−1.0	−1.0	−2.0 ± 1.8
	Net	−0.3	−0.3	−0.3	1.6		0.8	−0.8	2.3	−0.4	−1.7	−0.7	0.2	−0.8				−0.4	−1.6	−0.2	−0.2 ± 1.0
L4	In	0.3	n.a.	0.0	1.8	0.4	1.5	0.4	3.4	0.8	0.8	0.7	0.5	0.0	n.a.	1.4	1.5	0.1	n.a.	3.2	1.0 ± 1.0
	Ex	−1.1		−0.2	−0.9	−0.1	−1.7	−0.2	−4.2	−0.8	−1.0	−0.4	0.4	−0.1		−0.1	0.7	−0.7		−1.0	−0.7 ± 1.1
	Net	−0.8		−0.2	0.8	0.3	−0.2	0.2	−0.8	−0.1	−0.2	0.3	0.8	−0.1		1.3	2.2	−0.6		2.2	0.3 ± 0.9

Averaged across subjects^1^, C3/C5 = cervical levels 3/5, Th1/Th4/Th6/Th8/Th10 = thoracic levels 1/4/6/8/10, L2/L4 = lumbar levels 2/4, In = inspiration, Ex = expiration, Net = net volume; n.a. = not analyzed due to low SNR.

### ROI sizes during forced breathing

Because the flow volumes not only reflect flow velocities but also the underlying lumen (or chosen ROI), we further evaluated the size of the individual ROI as a function of time for all 19 subjects (see Supplementary Fig. 1). Although the values were highly variable both between subjects and along the spinal canal, the temporal evolution of ROI sizes revealed no distinct changes over time, not even during forced respiration. These results confirm that the observed alterations in CSF volumes are entirely evoked by changes of CSF flow velocities (see Fig. 2a) and not due to variations in ROI sizes.

## Discussion

Real-time phase-contrast flow MRI at high spatiotemporal resolution for the first time allowed for the evaluation of CSF dynamics along the entire spinal canal. We consistently found that forced inspiration is associated with an upward movement in the entire spinal canal in close agreement with our previous observations in the brain and upper spinal canal only. In contrast, forced expiration led to downward CSF flow below level Th6, but very low flow cranial to levels Th1 to Th4. This uniform pattern of CSF flow across subjects suggests a compartmentalization of the spinal subarachnoid space in such a way that CSF dynamics exhibit a watershed with a dividing point at about the level of the heart.

The occurrence of upward CSF flow into the head and brain in response to forced inspiration has been elucidated as a necessity to counterbalance the inspiratory-regulated venous outflow out of the head/neck region^12^. CSF and venous blood flow appear tightly interconnected and balanced to ensure the Monro-Kellie doctrine of a constant intracranial volume^9,13,14^. Based on the present results, this compensation of venous outflow from the brain during forced inspiration seems to constitute a remarkably strong regulator, which translates the upward surge of CSF from the cervical region along the entire spinal canal down to the lumbar thecal sac.

CSF flow during ensuing deep expiration in turn resulted in a pattern indicating pronounced caudal movements. The observed watershed-like pattern therefore draws attention on putative dependencies of CSF and venous flow in inferior regions of the spine. In fact, the large caval veins of thorax and abdomen as well as the vertebral plexus of the spine readily conform to external pressures. Respiration-induced changes of intrathoracic and intraabdominal pressures are transmitted via the abundant external venous plexus located in the paravertebral zones through the intervertebral foramina to the epidural spaces and their venous plexus therein. The intimate contact with the parietal pleura and the direct exposure to alternating pressures are shown by anatomic dissections^15–17^ and the pressure in the epidural space has been reported to oscillate with respiration^18,19^. Interestingly, a study of epidural pressures at different spinal segments revealed two opposing types of pressure changes in response to respiration^20^. In the upper body above the heart the pressure decreased during inspiration and rose during expiration, while in the lower abdominal lumbar areas pressures increased during inspiration and decreased throughout exhalation. Together, the spinal epidural space mirrored the pressure conditions in the superior caval venous system of the upper body and the inferior vena cava system of the lower body region^20^. Linking our present CSF findings more closely to these concepts would require parallel studies of lumbar epidural venous blood flow. However, such real-time flow MRI measurements are hampered by the variable and irregular local anatomy of the lumbar venous plexus and remain outside the scope of this study.

In conclusion, real-time flow MRI enables rapid and robust assessment of CSF velocities and volumes in the entire human spinal canal. This study revealed a watershed-like pattern of CSF movement during forced breathing where the divide could be identified at about the level of the heart. Forced inspiration distinctly perturbed that pattern and induced an upsurge of apparently the entire spinal CSF volume, whereas during the accompanying deep expiration the resting flow pattern was resumed. It can be postulated that the CSF flow counterbalances the effects of respiratory pressure changes in the tightly interconnected venous systems not only in the head and neck but also in the lower part of the body. These findings significantly expand our understanding of spinal CSF dynamics in humans. Translating real-time phase-contrast MRI to clinical applications will be of eminent importance to unravel the pathological mechanisms underlying spinal cord disorders such as syringomyelia and to open new options for therapies and respective evaluations.

## Methods

### Subjects

Twenty healthy volunteers were enrolled (age range 23–34 years, 6 females, 14 males) without contraindication for MRI and no known illness. The institutional review board of the Georg-August-University Goettingen approved the study and written informed consent was obtained from each subject before MRI. The study was in compliance with the Declaration of Helsinki.

### Study Design

CSF flow was quantitatively evaluated by real-time phase-contrast flow MRI along the entire length of the spinal canal. During MRI all subjects were placed in the supine position and required to follow a protocol (Fig. 1e) with 10 s of normal breathing followed by 4 cycles of 2.5 s forced inspiration and 2.5 s forced expiration, respectively, and again 10 s of normal breathing. The performance was monitored with use of a respiration belt. Moreover, adherence to the breathing protocol was verified by comparing the movement of the abdominal wall with the timing of the protocol and the observed CSF flow. Corresponding signal intensity changes (Fig. 1c) and CSF flow rates (Fig. 1d) in response to breathing demonstrate elevation and lowering of the abdominal wall as well as positive (i.e., upward) and negative (i.e., downward) CSF flow in association with inspiration and expiration.

### Real-Time MRI

All measurements were performed at 3 Tesla (Magnetom Prisma Fit; Siemens Healthcare). The real-time phase-contrast flow MRI technique^21–23^ is an extension of an advanced real-time MRI method^24^ which combines highly undersampled radial FLASH acquisitions with image reconstruction by regularized nonlinear inversion. Similar to conventional MRI studies of through-plane flow, the real-time technique involves the acquisition of two images with different velocity encodings perpendicular to the imaging section. The experimental parameters were: repetition time (TR) 5.7 ms, echo time (TE) 4.5 ms, slice thickness 5 mm, flip angle 10°, 1 × 1 mm^2^ in-plane resolution, field of view (FOV) 192 mm^2^ or 256 mm^2^. The two acquisitions with different velocity encodings employed 11 radial spokes each and provided a temporal resolution of 125 ms for the resulting pair of anatomic magnitude image and phase-contrast velocity map. Velocity sensitivity (VENC) varied between 10–40 cm s^−1^ and was adjusted according to CSF and epidural venous flow at the different levels of the spinal cord. While data at C3 were acquired with a 64-channel head coil, all other regions used suitable combinations of elements from the 18-channel thorax coil and 32-channel spine coil. Online reconstruction of real-time images was accomplished with use of a highly parallelized version of the reconstruction algorithm which was implemented on a computer with 8 graphical processing units (sysGen/TYAN Octuple-GPU, GeForce GTX TITAN, Nvidia; Sysgen, Bremen, Germany) bypassing the host of the MRI system in a fully integrated manner.

### Data Analysis

Analyses of real-time flow MRI measurements, i.e. serial anatomic images and velocity maps, was performed using CAFUR software (Fraunhofer Mevis, Bremen, Germany) specifically developed to perform automatic segmentation of structures in real-time MRI datasets^25^. Suitable ROIs were placed at cervical levels C3 and C5, thoracic levels Th1, Th4, Th6, Th8, Th10, and Th12, as well as lumbar levels L2 and L4. The definition of a ROI focused on all readily discernable flow signals observed in both the magnitude images and phase-contrast maps. Additional analyses were carried out using Matlab (Mathworks, USA).

### Data Availability Statement

The datasets generated during and analyzed during the current study are available from the corresponding author on reasonable request.

## Electronic supplementary material

Time course of ROI sizes for CSF analysis.
