# Supplementary material for: Respiration and the watershed of spinal CSF flow in humans
Source: Sci Rep. 2018 Apr 4;8:5594. doi: 10.1038/s41598-018-23908-z (PMC5884798; doi:10.1038/s41598-018-23908-z)
Supplement: Supplementary file 1 — Time course of ROI sizes for CSF analysis. [file 41598_2018_23908_MOESM1_ESM.docx]

**Respiration and the watershed of spinal CSF flow in humans**

Steffi Dreha-Kulaczewski^1*^, Mareen Konopka^2^, Arun A Joseph^3,4^, Jost Kollmeier^3^, Klaus-Dietmar Merboldt^3^, Hans-Christoph Ludwig^5^, Jutta Gärtner^1^, Jens Frahm^3,4^

^1^Department of Pediatrics and Adolescent Medicine, Division of Pediatric Neurology, University Medical Center Göttingen, 37075 Göttingen, Germany. ^2^School of Medicine, University Medical Center Göttingen, 37075 Göttingen, Germany. ^3^Biomedizinische NMR Forschungs GmbH am Max-Planck-Institut für biophysikalische Chemie, 37077 Göttingen, Germany. ^4^DZHK (German Center for Cardiovascular Research), partner site Göttingen, Germany. ^5^Department of Neurosurgery, Division of Pediatric Neurosurgery, University Medical Center Göttingen, 37075 Göttingen, Germany.

**Supplementary Figure 1.** Time course of ROI sizes for CSF analysis.
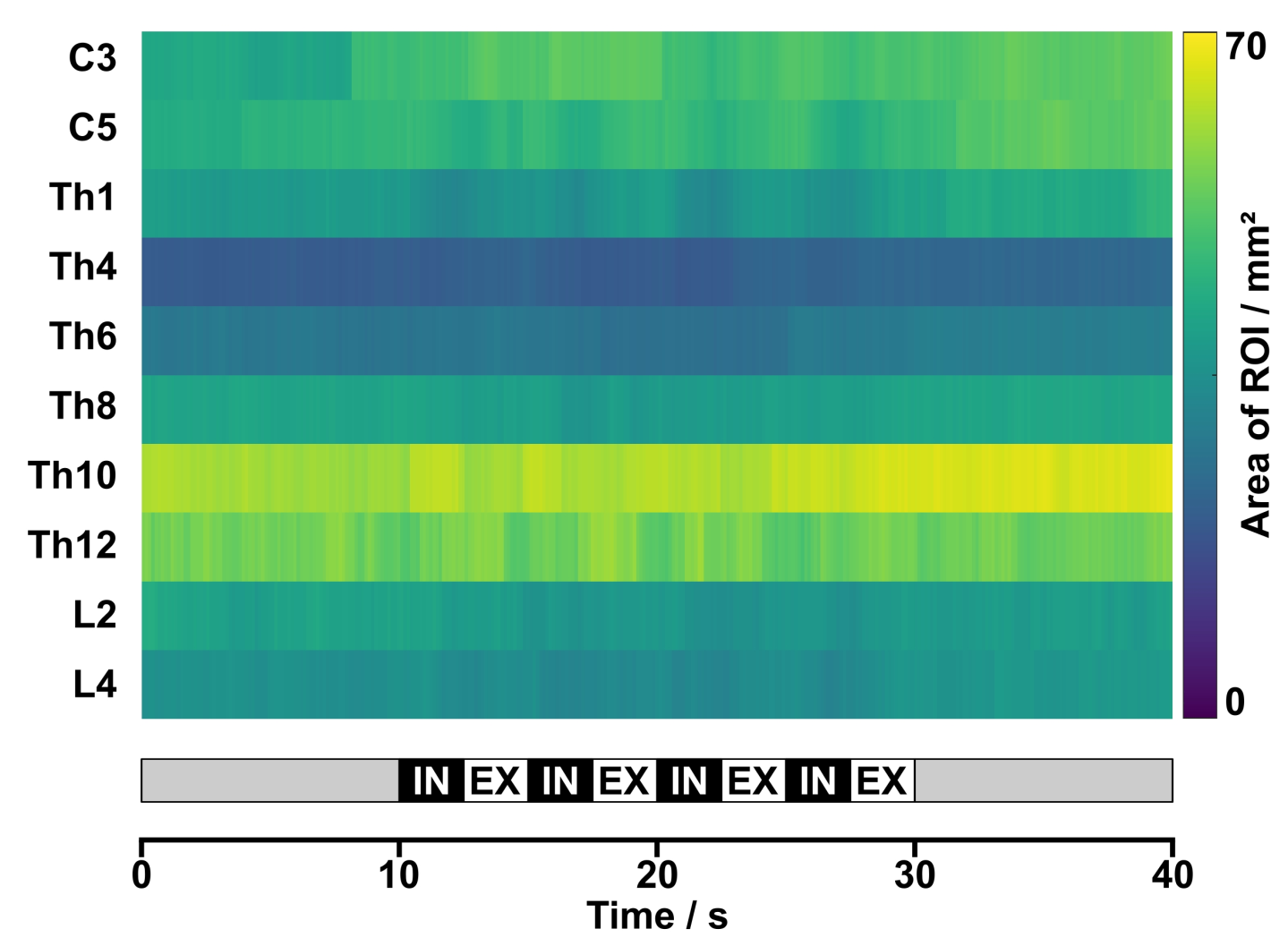


**Supplementary Figure 1.** Time course of ROI sizes for CSF analysis. Color-coded mean areas (mm^2^) averaged across subjects show no significant change over time, in particular not during forced breathing: IN = inspiration, EX = expiration.
